# Do Brand Competence and Warmth Always Influence Purchase Intention? The Moderating Role of Gender

**DOI:** 10.3389/fpsyg.2020.00248

**Published:** 2020-02-21

**Authors:** Jianping Xue, Zhimin Zhou, Liangbo Zhang, Salman Majeed

**Affiliations:** ^1^College of Management, Shenzhen University, Shenzhen, China; ^2^College of Economics and Management, Yibin University, Yibin, China

**Keywords:** brand perception, moderating role, gender, brand trust, purchase intention, experimental method

## Abstract

Consumers’ perceptions of a brand (e.g., competence or warmth) may directly affect their brand trust, purchase intention, and ability to achieving corporate goals of sustainability. However, gender acts as a moderator in the influence of brand perception on purchase intention. This study examined the main effects of brand perception on purchase intention, the moderating effect of gender, and the mediating effect of brand trust by conducting two experiments and a path analysis. Findings from experiment 1 show that perceived warmth and perceived competence exert significant positive effects on purchase intention with brand trust as a mediator. Findings from experiment 2 indicate that perceived warmth is influential only for female customers, not for male customers, that is, gender moderates the relationship between perceived warmth and purchase intention. However, gender does not moderate the influence of perceived competence on purchase intention. The results of the path analysis are consistent with the experimental results, indicating that the conclusions of the study are robust and reliable. Finally, theoretical contributions and managerial implications are discussed.

## Introduction

Brand management, a key marketing strategy for enterprises (Capron and Hulland, 1999; Ertimur and Coskuner-Balli, 2015), includes the management of explicit elements, such as brand name and logo (Bresciani and Ponte, 2017; Luffarelli et al., 2019), and management of implicit elements, such as brand relationships and brand personality (Aaker, 1997; Machado et al., 2019). The entirety of these elements can influence the psychology and behavior of customers and may ultimately affect enterprises’ brand equity (Bairrada et al., 2019; Bettels and Wiedmann, 2019). Many brand-related studies demonstrate that brands have specific characteristics similar to human personalities and the relationships between people and brands resemble interpersonal relationships (Aaker, 1997; Aggarwal, 2004; Grohmann, 2009). In addition, consumers can execute brand evaluation in an identical approach to how they assess groups or individuals, thus forming a specific brand impression similar to personality traits (Portal et al., 2018). Social psychologists propose the stereotype content model describing how individuals evaluate a social group or an individual and form their own social judgments on the group or the individual (Fiske et al., 2002). In this aforementioned model, warmth and competence constitute the model’s two primary dimensions, with each being divided into high and low levels; accordingly, four types of social judgment are formed after pair-wise combination. Marketing scholars have successfully introduced the model into the field of brand research and used theory to describe consumers’ specific perceptions of brands (Aaker et al., 2012; Kervyn et al., 2012). Kervyn et al. (2012) propose brands as intentional agents framework, which can explain consumers’ perceptions of brands. Perceptions can trigger different emotions and brand behaviors. Thus, brands as the intentional agents framework are very useful for brand positioning and brand communication. Such perceptions, also referred to as “brand perceptions” by the scholars, are individuals’ social judgments of a brand based on their own specific impressions of the concerned brand (Chan et al., 2018; Portal et al., 2018). Brand warmth can be defined as consumers’ perceptions of a brand’s good intentions, and it emphasizes on a brand’s friendliness, genuineness, and helpfulness. Brand competence can be defined as consumers’ considerations that a brand has the ability and skills to meet consumers’ intentions, and it emphasizes on a brand’s competitiveness, intelligence, and skills (Judd et al., 2005; Cuddy et al., 2007). It is noted that consumers’ perceptions of a brand’s warmth and competence influence their purchase intentions and brand loyalty (Kervyn et al., 2012), admiration (Aaker et al., 2012), attitude (Ivens et al., 2015), and trust (Kim et al., 2018). Purchase intention is a core construct of consumer behavior and is defined as subjective likelihood that consumers will buy a specific product (Dodds, 1991). Consumers believe that a brand’s products will be desirable if the brand is perceived as a highly competent brand which may improve consumers’ purchase intentions for products (Aaker et al., 2012). However, in case of product failure, perceived warmth has greater predictive power for purchase intention than does perceived competence (Zawisza and Cinnirella, 2010; Xu et al., 2013). Recent research shows that perceived warmth and perceived competence are both important drivers of purchase intention (Kolbl et al., 2019; Crisafulli et al., 2020). Technology anxiety, people-focused advertising versus self-focused advertising appeal types, and product involvement moderate the influence of warmth and competence on the purchase intention of consumers (Zawisza and Pittard, 2015). Consumers consider various channels to obtain brand information, purchase brand-related products, use various services provided by brands, and participate in brand-related activities in tandem with gradually forming a brand impression as their understanding of the brand deepens (Schnurr, 2017). Scholars note that significant differences exist in the information-processing methods between male and female consumers (Darley and Smith, 1995). According to the empathizing–systemizing theory, men’s psychology and behaviors are more influenced by cognition, while women are more affected by emotion. Accordingly, gender may have moderating effects on the influence exerted by brand perception on purchase intention because of these differences. This study focused on answering three research questions by conducting two experiments and a path analysis: (1) Can both perceived warmth and competence always stimulate purchase intention of consumers? (2) What mediating mechanism is underlying competence and warmth’s effect on this purchase intention? and (3) Do perceived competence and warmth’s effects on purchase intention vary between genders?

## Literature Review

### Brand Perception

The concept of brand perception originates from the stereotype content model (Aaker et al., 2012), which portrays people’s social judgments of groups or individuals through two basic dimensions (Fiske et al., 2002), i.e., (1) warmth, including sincerity, friendliness, helpfulness, and enthusiasm; and (2) competence, including ability, efficiency, skill, and self-confidence (Aaker et al., 2010). The use of brand perception is highly prevalent in many fields, including advertising, sensory marketing, and corporate image. Sensory marketing literature indicates that the angular and circular cues of a service environment can motivate the perceptions of service providers’ competence and warmth among consumers, respectively, thereby increasing customer satisfaction (Liu et al., 2018). Furthermore, online advertising and offline advertising affect consumers’ brand perceptions with respect to warmth and competence, respectively; such perceptions can also influence purchase intention (Zawisza and Pittard, 2015; Chan et al., 2018). However, competence may exert a significant effect on consumers’ purchase intention (Aaker et al., 2012). Additionally, a previous study suggests that consumers perceive non-profit organizations to be relatively warm albeit less competent, which reduces their purchase intention; by contrast, organizations with high warmth and high competence promote feelings of admiration among consumers alongside enhancing their purchase intention (Aaker et al., 2010). The intensity of marketers’ smiles in advertising can prompt consumers to develop associations with the marketers with greater warmth or competence, thereby increasing purchase intention (Wang et al., 2017). In the business to business setting, both competence and warmth affect purchase intention (Crisafulli et al., 2020). Thus, literature review reveals inconsistency in conclusions pertaining to the influence exerted by perceived warmth on consumers’ purchase intention. Perceiving a particular brand to be of high competence can advance consumers’ positive evaluation of product quality and performance, thus further increasing their purchase intention. In addition, perceiving a particular brand to have high warmth can promote consumers’ positive evaluations of the relevant enterprise and brand as sincere, friendly, and even considerate. When customers feel fully respected and understood by an enterprise or brand, their needs in relation to respect and acceptance – as per Maslow’s hierarchy of needs theory – are met. This further promotes consumers’ positive attitudes toward the enterprise and brand; consequently, their purchase intention is influenced significantly and positively. Therefore, in the current study, the following hypotheses were proposed:

H_1_: Perceived warmth exerts a significant and positive effect on purchase intention.
H_2_: Perceived competence exerts a significant and positive effect on purchase intention.

### Mediation Effect of Brand Trust

The brand personification and personality literature indicates that brands possess characteristics similar to those of people, and the relationships between consumers and brands resemble interpersonal relationships (Swaminathan et al., 2007). Therefore, psychological mechanisms underlying brand trust among consumers should be comparable to those underlying interpersonal relationship trust (Fournier, 1998). Brand trust among consumers is conducive to reducing the risks consumers perceive in relation to a brand in the purchasing process. Brand trust increases consumer confidence in a brand and its products, promoting consumer confidence in the brand’s performance, honesty, and kindness. In other words, when consumers perceive a particular brand to be warm, they exhibit a relatively high likelihood of trusting in the brand’s honesty and kindness (Kim et al., 2018); brands that consumers perceive to be competent have a relatively high likelihood of gaining consumers’ trust with respect to their competence. Brand trust results from consumer brand cognition and brand experience, reflecting the emotional relationship that exists between brands and consumers; this signifies that consumers’ brand perceptions should be the premise of consumer brand trust. Regarding its establishment, brand trust is gradually formed in the process of the accumulation of brand knowledge and brand experience; the brand competence perception of consumers strongly and positively affects brand trust (Sung and Kim, 2010). From a psychological perspective, consumers’ trust in brand can influence their decision making in purchase choices, i.e., high trust level leads to fewer brand comparisons with favorable purchase decisions and vice versa. This also saves psychological resources in consumers’ decision making in parallel to increasing the likelihood of consumer purchasing behavior. Accordingly, in the current study, we introduced the following hypotheses:

H_3_: Brand trust mediates the influence of perceived warmth on purchase intention.
H_4_: Brand trust mediates the influence of perceived competence on purchase intention.

### Moderating Effect of Gender

Gender is an influential variable in consumer behavior research. Gender differences have been observed with respect to information processing and behavioral responses. From a psychological perspective, the empathizing–systemizing theory holds that there are significant gender differences in empathizing and systemizing, that is, women’s psychology and behavior are more emotionally dominated, while men are more cognitively dominated (Baron-Cohen, 2016). Evidence from neurobiology shows that women’s trust depends on emotions and men’s trust depends on cognition, which is consistent with the empathizing–systemizing theory (Riedl et al., 2010). Research related to e-commerce has found that women are more likely to find cues to details and emotionally affect their trust (Rodgers and Harris, 2003). In conventional buying, research has found that emotional factors are more important than functional concerns for women, but the opposite is true for men (Dittmar et al., 2004). From the perspective of information processing, there are significant differences in the way men and women handle information. Some studies have suggested that men tend to handle advertising information in a heuristic manner (Kempf et al., 2006; Papyrina, 2015), and the relationship between the number of arguments and persuasiveness of information is positive and linear. Regarding the reaction of women to advertising information, a non-linear relationship exists between the number of arguments and effectiveness of advertising. Women adopt a more detailed and selective approach with respect to advertising information (Papyrina, 2019). Additionally, research demonstrated women to be thorough processors of information in that they analyze objective as well as subjective product attributes; by contrast, the same research demonstrated men to be selective processors of information in that they exhibit a tendency to heuristically process information and concurrently ignore subtle cues (Darley and Smith, 1995). Kempf et al. (2006) note men to exhibit a tendency of applying off-the-shelf information to establish brand judgments; this explains their relatively low likelihood of perceiving other attribute information in the course of product trials. Thus, the aforementioned studies have consistently concluded that men use heuristic strategies to process information and that this process is selective, whereas women process information in more detail and devote more attention to small cues. Trust and purchase intentions of men are more affected by cognitive components, while women are more affected by emotional components. Accordingly, considering the preceding specifications, we derived the following hypotheses in this study:

H_5_: Gender exerts its moderating impact on the relationship between perceived competence and purchase intention; more precisely, the perceived competence generates more purchase intentions for male than for female.
H_6_: Gender exerts its moderating impact on the relationship between perceived warmth and purchase intention; more precisely, perceived warmth generates more purchase intentions for female than for male.

**FIGURE 1:**
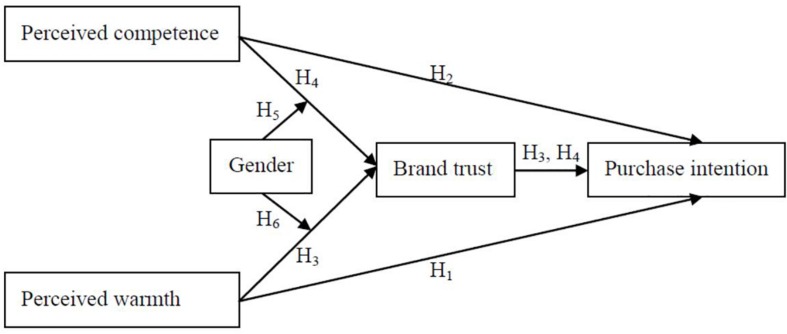
Research model.

In the current study, we conducted two experiments and a path analysis to probe brand perception’s main effects on purchase intention as well as brand trust’s mediating effects and gender’s moderating effects. The research model is illustrated in Figure 1.

## Experimental

### Pre-experiment

The pre-experiment was conducted with the purpose of testing the discrimination of brand perception stimuli. The two formal experiments required two stimuli that may stimulate perceptions of competence and warmth among consumers; we considered various perception stimuli to ensure the robustness of the conclusions. The pre-experimental procedure comprised of three steps: First, brand perception stimulus materials and experimental brand perception questionnaires were designed through discussion with marketing professors. The measurement scales of competence and warmth, each containing four items, were adopted from the studies of Aaker et al. (2010) and Wang et al. (2017). Perceived warmth is related to sincerity, friendliness, warmth, and enthusiasm, whereas perceived competence is related to competence, efficiency, skill, and confidence. Second, pre-testing was performed by administering brand perception questionnaires to colleagues and friends in order to investigate whether they could easily read and understand the stimulus materials to be used in the experiments. We subsequently modified the materials according to the thorough feedback and finally designed two types of pre-experimental stimulus materials related to brand perception, namely (1) stimulus materials that portrayed brand A as competent and (2) stimulus materials that portrayed brand A as warm. Items related to the stimulus materials that portrayed brand A as competent are outlined as follows: ‘Brand A has products with numerous original design patents displaying excellent performance and fine workmanship’; ‘Brand A has strong product research and development capabilities, far exceeding those of other competitors in the industry’. Additionally, items related to the stimulus materials that portrayed brand A as warm are outlined as follows: ‘Brand A produces products with an excellent word-of-mouth reputation and user-friendly product design’; ‘Brand A provides customers with meticulous service and advocates a customer-first approach, making customers feel that the brand is consistently supportive’. Third, the revised stimulus materials of brand perception were uploaded on *Wenjuan Xing*^1^, an online academic survey website in China, to conduct an online pre-experiment. In particular, an intergroup experiment was used to prevent cross-interference between competence and warmth perception materials. Of 37 valid questionnaires for the ‘competence perception group’, 30 were from female respondents; of 31 valid questionnaires for the ‘warmth perception group’, 25 were from female respondents. Respondents were shown one of the two brand perception stimulus materials and were asked to rate, on a 7-point Likert scale, four items used for measuring competence perception (e.g., ‘Brand A is sincere’: 1 = *strongly disagree* and 7 = *strongly agree*; Cronbach α = 0.84) and four items used for measuring warmth perception (e.g., ‘Brand A is efficient’: 1 = *strongly disagree* and 7 = *strongly agree*; Cronbach α = 0.88). Finally, they were asked questions related to demographic characteristics. A cross-table analysis and χ^2^ test revealed the competence and warmth groups to exhibit no significant differences with respect to demographic characteristics; in other words, the members of the two groups potentially exhibited similar demographic characteristics. The study used a paired-sample t test to analyze brand perception differences for the two sets of materials corresponding to perceived competence and perceived warmth. The brand perception measurement result (M_competence_ = 5.65 vs. M_warmth_ = 4.77, *t* = −5.971, df = 36, *p* < 0.01) indicated that for the competence stimuli, perceived competence significantly exceeded perceived warmth. The brand perception measurement result (M_warmth_ = 5.54 vs. M_competence_ = 4.66, *t* = 4.13, df = 30, *p* < 0.01) indicated that for the warmth stimuli, perceived warmth significantly exceeded perceived competence. Differences in competence and warmth perceptions for the two sets of materials were also analyzed. Perceived competence was significantly higher for the competence perception material than it was for the warmth perception material (M_competence–competence material_ = 5.65 vs. M_competence–warmth material_ = 4.66, *t* = 4.945, df = 66, *p* < 0.01). Moreover, perceived warmth was significantly higher for the warmth perception material than it was for the competence perception material (M_warmth–warmth material_ = 5.54 vs. M_warmth–competence material_ = 4.77, *t* = −3.12, df = 66, *p* < 0.01). Therefore, the two sets of stimulatory materials exhibited significant brand perception differences, indicating that the design of the stimulatory materials effectively discriminated between warmth and competence perceptions. The competence and warmth materials were thus used in Experiments 1, Experiments 2, and path analysis.

### Experiment 1

#### Purposes and Procedures

In this experiment, we probed (1) brand perception’s main effects on purchase intention and (2) brand trust’s mediating effect on the brand perception–purchase intention relationship. An online experiment was also conducted on the *Wenjuan Xing* website by using the competence material from the pre-experiment as the stimulus. A virtual brand called ‘Albern’ was fabricated for the experiment to overcome the inherent stereotypes of consumers regarding real brands. The main reason for using an English version of the brand name rather than a Chinese version was to prevent interference of and confusion with any other brand in Chinese. To confirm this, before the experiments, *Baidu*, one of the most popular search engines in China, was used to search for the ‘Albern’ brand name, and no identical brand name appeared in the results. After consultation with a marketing professor, a loudspeaker box selected from among 15 stimulus products was included for use. The experiment first required the participants to watch competence materials for the ‘Albern’ loudspeaker box and to provide ratings on relevant scales. The participants were then asked questions on their demographic characteristics.

#### Participants and Measurement

This online experiment had 148 respondents, with all questionnaire responses being valid. There were 91 female respondents. Their age range was 15–64 years (M_age_ = 32.2, standard deviation [SD] = 8.24 years). Ninety-five percent of the participants were determined to have received higher education and above. The participants were shown the competence perception stimulus materials from the pre-experiment and asked to respond to four items used for measuring competence perception (e.g., ‘Brand Albern is sincere’: 1 = *strongly disagree* and 7 = *strongly agree*; Cronbach α = 0.65) and to four items used for measuring warmth perception (e.g., ‘Brand Albern is efficient’: 1 = *strongly disagree* and 7 = *strongly agree*; Cronbach α = 0.63). Purchase intention was measured on a 7-point Likert scale using three items (e.g., ‘There was high probability that I would consider buying this brand’s product’: 1 = *strongly disagree* and 7 = *strongly agree*; Cronbach α = 0.76); moreover, brand trust was measured on a 7-point Likert scale using four items (e.g., ‘I trust this brand’: 1 = *strongly disagree* and 7 = *strongly agree*; Cronbach α = 0.76) (Grewal et al., 1998; Chaudhuri and Holbrook, 2001). Finally, the participants were asked questions regarding their demographic characteristics.

#### Main Effect

We conducted regression analysis in this study to probe the main effects exerted by brand perception on purchase intention; in addition, both competence perception and warmth perception were incorporated into the regression equation: The derived results are outlined as follows: *R*^2^ = 0.39, *F*(2,145) = 45.69, and *p* < 0.01, signifying brand perception to exert a significant main effect. In other words, brand perception was determined to exert a significant predictive effect on purchase intention. Competence and warmth perceptions were both determined to exhibit a significant positive effect on purchase intention (*B* = 0.54 and 0.29, respectively; *t* = 6.03 and 2.97, respectively; both *p* < 0.01); accordingly, hypotheses H_1_ and H_2_ were determined to be supported. According to the preceding data analysis results, the regression coefficient for competence perception was greater than that for warmth perception, indicating that competence perception has more influence on purchase intention than warmth perception does.

#### Mediating Effect

This study adopted the moderated mediation method of Hayes (2018) for mediation analysis. The two perception types were set as the independent variables, whereas brand trust and purchase intention were set as the mediator and dependent variables, respectively. The study analyzed the mediating role of brand trust alone. Model 4 was used, the bootstrap sample size was set to 5000, and the confidence interval (CI) was set to 95% (i.e., the CI did not contain 0, indicating that brand trust constituted the mediator in the influence exerted by brand perception on purchase intention). With perceived competence as an independent variable, the mediation analysis results were as follows: β = 0.34, SE = 0.07 and 95% CI = 0.22–0.48, indicating that the mediating effect of brand trust was significant. After this mediating effect was eliminated, perceived competence was determined to exert a significant direct effect on purchase intention (β = 0.34, SE = 0.77, *t* = 4.48, 95% CI = 0.19–0.49, and *p* < 0.01), indicating brand trust to have a partial mediating influence on perceived competence’s effect on purchase intention. With perceived warmth as an independent variable, the mediation analysis results were as follows: β = 0.49, SE = 0.07 and 95% CI = 0.35–0.63, indicating that brand trust’s mediating effect was significant. After this mediating effect was eliminated, perceived warmth’s direct effect on purchase intention was insignificant (β = 0.12, SE = 0.09, *t* = 1.31, 95% CI = −0.06 to 0.31, and *p* = 0.19), signifying that brand trust completely mediated perceived warmth’s effect on purchase intention. Therefore, the mediation analysis results were determined to support hypotheses H_3_ and H_4_; in other words, brand trust mediates brand perception’s influence on purchase intention. Brand trust was determined to exhibit different degrees of mediation of the influence of perceived competence and perceived warmth on purchase intention: partial and complete mediation.

### Experiment 2

#### Purposes and Procedures

This experiment had two purposes. First, by using different experimental materials, we re-examined brand perception’s main effect and brand trust’s mediating effect to ensure robust conclusions. Second, after the influence of consumer involvement was controlled for, the moderating effect of gender was examined. The warmth perception materials in the pre-experiment were used as stimuli in Experiment 2. Nevertheless, the brand name remained ‘Albern’; moreover, the experimental stimulation products and experimental procedures remained identical to those in Experiment 1. As per the pre-experiment, the consumers’ perceived competence associated with the material might be relatively low, whereas the perceived warmth associated with the materials might be relatively high. In Experiment 2, we used different materials from those in Experiment 1 to determine whether the material type affected the research conclusions, helping to ensure the robustness of the Experiment 1 results. Previous research found different responses to low-involvement product advertising between genders (Garlin and McGuiggan, 2002; Papyrina, 2015). Competence has been shown to engender increased purchase intention in high-involvement products (Aaker et al., 2010; Zawisza and Pittard, 2015). From the perspective of consumers, consumer involvement is more meaningful than product involvement (Zaichkowsky, 1985). Different consumers devote different types of attention to the same product purchase decision, and their input energy levels differ because of their different involvement levels. Consumer involvement affects information processing, so that both gender and consumer involvement may moderate brand perception’s effects exerted on purchase intention (Zawisza and Pittard, 2015). Thus, the effects of consumer involvement were controlled and examined gender’s moderating effect alone. The online experiment was conducted on the *Wenjuan Xing* website.

#### Participants and Measurement

In total, 146 valid responses were received. There were 77 female respondents. Respondents’ age range was 18–53 years (*M*_age_ = 31.51, SD = 6.98 years). Ninety-eight percent of the participants were determined to have received higher education and above. The participants were shown the warmth perception stimulus materials from the pre-experiment and asked to answer the corresponding items on some relevant scales, including items for competence perception (Cronbach α = 0.66), warmth perception (Cronbach α = 0.64), brand trust (Cronbach α = 0.68), and purchase intention (Cronbach α = 0.71). These scales were identical to the scales used in Experiment 1. The participants were also asked to respond to six items on a 7-point Likert scale in order to evaluate their consumer involvement in buying the loudspeaker box (e.g., ‘Buying this product is very important to me’: 1 = *strongly disagree* and 7 = *strongly agree*; Cronbach α = 0.79) (Traylor and Joseph, 1984; Zaichkowsky, 1985). Finally, the participants were asked to answer questions regarding their demographic characteristics.

#### Main Effect

We conducted regression analysis in this study to probe the main effect exerted by brand perception on purchase intention; both perceived competence and perceived warmth were inserted into the regression equation. The regression analysis results were as follows: *R*^2^ = 0.38, *F*(2,143) = 42.98, and *p* < 0.01, indicating brand perception to still exhibit a significant main effect under the stimulation of warmth materials; in other words, brand perception still demonstrated a significant predictive effect toward purchase intention. Perceived competence (*B* = 0.42, *t* = 5.52, and *p* < 0.01) and perceived warmth (*B* = 0.36, *t* = 4.48, and *p* < 0.01) were noted to exert significant positive effects on purchase intention; accordingly, hypotheses H_1_ and H_2_ were determined to be supported. Data analysis also suggested that the regression coefficient for perceived competence remained greater than that for perceived warmth perception, demonstrating that perceived competence influences purchase intention more than perceived warmth does. In summary, the findings of Experiments 1 and 2 are consistent, indicating a robust and reliable main effect of brand perception on purchase intention.

#### Mediating Effect

Consumers typically exhibit greater willingness to invest energy and time in comparing schemes or collecting data when making high-involvement purchase decisions than they do when making low-involvement purchase decisions. The elaboration likelihood model shows that a central path is used by consumers to process information for high-involvement purchase decisions, making decisions through systematic thinking. By contrast, they use peripheral paths to process information for low-involvement purchase decisions, making decisions through heuristic thinking. Therefore, consumer involvement, a consumer purchasing decision key variable, may interfere with the brand perception–purchase intention relationship. The correlations of consumer involvement with brand trust (*r* = 0.63, *p* < 0.01) and consumer involvement with purchase intention (*r* = 0.66, *p* < 0.01) were significant and positive. Therefore, consumer involvement was set as a covariate of the moderated mediation model to control for its effects on purchase intention. Additionally, consumer involvement was set as the covariate; the other analysis parameters were the same as those for Experiment 1. The analysis results revealed brand trust to still exhibit a mediating effect. After brand trust’s mediating effect was eliminated, perceived warmth and perceived competence had no direct effects on purchase intention. After consumer involvement’s effect exerted on purchase intention was controlled for, competence perception was determined to have a significant indirect effect on purchase intention (β = 0.16, SE = 0.05, and 95% CI = 0.08–0.26); in other words, brand trust’s mediating effect was significant. After this mediating effect was eliminated, the direct effect of perceived competence on purchase intention was insignificant (β = 0.11, SE = 0.07, *t* = 1.48, 95% CI = −0.04 to 0.26, and *p* = 0.14), indicating that brand trust completely mediated perceived competence’s effect on purchase intention. After the consumer involvement’s effect was controlled for, perceived warmth was noted to exert a significant indirect effect on purchase intention (β = 0.15, SE = 0.06, and 95% CI = 0.05–0.27); in other words, brand trust’s mediating effect remained significant. After brand trust’s mediating effect was eliminated, perceived warmth’s direct effect on purchase intention remained insignificant (β = 0.08, SE = 0.08, *t* = 1.06, 95% CI = −0.07 to 0.23, and *p* = 0.29), indicating that brand trust is a complete mediator of perceived warmth’s effect on purchase intention. After consumer involvement’s effect was controlled for, brand trust’s mediating effects remained, supporting hypotheses H_3_ and H_4_ and indicating that Experiment 1’s results are robust and not subject to interference from consumer involvement.

#### Moderated Mediation Effect

Gender differences may result in differences in information processing. Women notice subtle cues more and tend to comprehensively process information, whereas men often selectively process information and adopt heuristic thinking. Therefore, brand perception’s influence on purchase intention may be moderated by gender. The analysis method in Experiment 2 was identical to that used in Experiment 1, but with gender as a moderator; Model 8 was selected, and consumer involvement was set as a covariate. The other parameters were the same as those in Experiment 1. First, we analyzed gender’s moderating effects on the influence exerted by perceived competence on purchase intention. For male consumers, brand trust had a significant mediating effect (β = 0.15, SE = 0.06, and 95% CI = 0.04–0.26). After this mediating effect was eliminated, perceived competence was noted to exert a significant direct effect on purchase intention (β = 0.21, SE = 0.09, *t* = 2.22, 95% CI = 0.02–0.40, and *p* = 0.03). Therefore, for male consumers, brand trust partly mediated perceived competence’s influence on purchase intention. For female consumers, brand trust was determined to exhibit a significant mediating effect (β = 0.17, SE = 0.05, and 95% CI = 0.08–0.29), whereas after this mediating effect was eliminated, perceived competence was observed to have an insignificant effect on purchase intention (β = 0.06, SE = 0.09, *t* = 0.16, 95% CI = −0.17 to 0.20, and *p* = 0.87). Therefore, for female consumers, brand trust completely mediated perceived competence’s influence on purchase intention. Furthermore, for both female and male consumers, perceived competence was determined to affect purchase intention, with the mediator being brand trust. Regarding the influence of perceived competence on purchase intention, gender was noted to exhibit no significant moderating effect. Thus, hypothesis H_5_ was not supported. Second, we probed gender’s moderating effect on the perceived warmth–purchase intention relationship. For male consumers, perceived warmth’s direct effect on purchase intention was insignificant (β = 0.08, SE = 0.1, *t* = 0.8, 95% CI = −0.11 to 0.26, and *p* = 0.43); the mediating effect of brand trust was insignificant (β = 0.07, SE = 0.07, and 95% CI = −0.05 to 0.22). Thus, perceived warmth did not influence the men’s purchase intention. For female consumers, perceived warmth’s direct effect on purchase intention was insignificant (β = 0.09, SE = 0.11, *t* = 0.81, 95% CI = −0.12 to 0.29, and *p* = 0.42), whereas brand trust’s mediating effect was significant (β = 0.24, SE = 0.06, and 95% CI = 0.12–0.37). Thus, warmth perception effectively increased the women’s purchase intention. The analysis results demonstrate perceived warmth’s indirect effect on purchase intention: perceived warmth significantly influenced women’s purchase intention through brand trust, whereas perceived warmth’s effect on purchase intention in men was insignificant. Therefore, gender exerts a significant moderating effect regarding perceived warmth’s influence on purchase intention, supporting hypothesis H_6_.

## Structural Equation Modeling (Sem) for Path Analysis

### Data Analysis Strategy

The main purpose of conducting the path analysis is to verify if gender has a moderating role in the relationship between perceived warmth and brand trust to confirm the above conclusions and to ensure the robustness of the conclusions. For sufficient sample size, the study used the data collection method of Experiment 2 to supplement 417 valid samples, which yield the total number of samples to 563. Respondents’ age ranged from 18–64 years (*M*_age_ = 30.23, SD = 7.6 years). There were 300 female respondents. Approximately 97% respondents declared higher education and above. This study used Partial least square (PLS) to validate the research model because it is a suitable method to analyzing the structural relationship of latent variables (Chin and Newsted, 1999), categorical moderation, and mediation analysis (Hair et al., 2016; Wong, 2016; Nitzl and Chin, 2017). Therefore, in terms of data analysis strategy, smart PLS 3.2.7 was adopted to test the research model.

### Measurement Model

All latent variables are reflective first-order construction. The reliability of the measurement model was first assessed. Findings show that factor loading for all indicators ranges from 0.735 to 0.907, and are acceptable (Chin, 1998). The composite reliability (CR) of all constructs ranges from 0.897 to 0.932, indicating internal reliability (Nunnally and Bernstein, 1994). Results are summarized in Table 1.

**TABLE 1:** Assessment of the measurement model.

Items	Loading
**Perceived competence (CR = 0.897; AVE = 0.687)**	
Brand Albern is competent;	0.888
Brand Albern is efficient;	0.844
Brand Albern is skillful;	0.842
Brand Albern is confident;	0.735
**Perceived warmth (CR = 0.9; AVE = 0.693)**	
Brand Albern is sincere;	0.822
Brand Albern is friendly;	0.856
Brand Albern is warm;	0.830
Brand Albern is enthusiastic;	0.821
**Brand trust (CR = 0.927; AVE = 0.761)**	
I trust this brand;	0.885
I rely on this brand;	0.881
This is an honest brand;	0.885
This brand is safe;	0.838
**Purchase intention (CR = 0.93; AVE = 0.815)**	
I was interested in buying this brand‘s product;	0.907
I was satisfied with this brand’s product in general.	0.896
There was high probability that I would consider buying this brand’s product;	0.905
**Consumer involvement (CR = 0.932; AVE = 0.696)**	
Buying this product is very important to me;	0.847
Buying this product is closely related to me;	0.842
I am very concerned about purchasing this product;	0.876
This product is valuable;	0.813
This product is useful;	0.776
I am interested in this product;	0.847

*CR = composite reliability, AVE = average variance extracted.*

Second, the validity of the measurement model was assessed. The average variance extracted (AVE) is used to evaluate the convergent validity of items and the lowest threshold limit is usually 0.5 (Fornell and Larcker, 1981). The AVE of all constructs measurement far exceeds the threshold limit of 0.5 and ranges from 0.687 to 0.815, indicating that items of each construct have better convergent validity (see Table 1). Subsequently, the discriminate validity between the constructs based on the Fornell-Larcker criterion is evaluated. The AVE square roots of each construct are greater than their correlation coefficients with other constructs, indicating that the discriminate validity between constructs is adequate (Fornell and Larcker, 1981). These results are presented in Supplementary Material.

### Structural Model Evaluation

**FIGURE 2:**
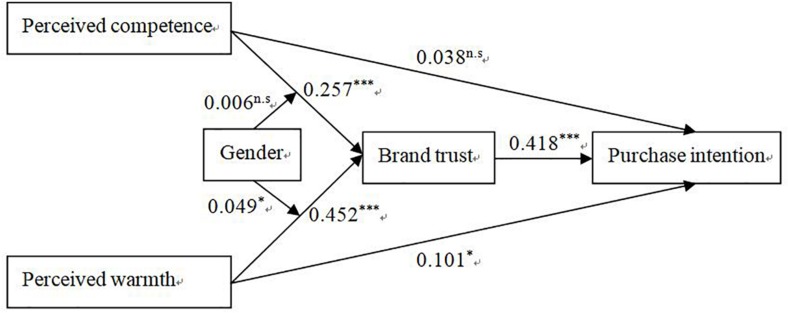
Path coefficient model. ^∗^*p* < 0.05, ^∗∗^*p* < 0.01, ^∗∗∗^*p* < 0.001, ^*n.s*^*P* > 0.05.

Path analysis was used to test the hypotheses proposed in the research model. A bootstrapping procedure was performed. Consumer involvement, age, and education were considered as control variables in the model to avoid their effects. Gender was set as a moderator, with a male assignment of 1 and a female assignment of 2. The test results of the research hypotheses are presented in Table 2. Findings show that the hypothesis of testing the research model using the path analysis is consistent with the conclusion of the experimental method. In terms of total effects, perceived warmth significantly and positively affects purchase intention (β = 0.29, *t* = 5.972), supporting hypothesis H_1_. Perceived competence also have a significant and positive impact on purchase intention (β = 0.145, *t* = 2.682), supporting hypothesis H_2_. In terms of the mediating effect, both perceived warmth (β = 0.189, *t* = 6.186) and perceived competence (β = 0.107, *t* = 4.293) significantly affect purchase intentions through brand trust as a mediator, supporting the hypotheses H_3_ and H_4_. In terms of moderating effects, the moderating effect of gender is not significant in the relationship between perception competence and purchase intention (β = 0.002, *t* = 0.265). Thus, hypothesis H_5_ is rejected. However, findings show that gender significantly moderates the relationship between perceived warmth and purchase intention (β = 0.021, *t* = 2.009), supporting hypothesis H_6_, that is, brand warmth is more likely to promote female consumers’ purchase intentions than male consumers. Figure 2 shows path coefficients of the structural equation model. Total effect, mediation effect, and moderating effect can be measured by calculating the corresponding path coefficients.

**TABLE 2:** Research hypothesis results.

Hypotheses	Relationship	Effect value	*t*-Value	Results
H_1_	PW- > PI	0.290***	5.972	Supported
H_2_	PC- > PI	0.145**	2.682	Supported
H_3_	PW- > BT- > PI	0.189***	6.186	Supported
H_4_	PC- > BT- > PI	0.107***	4.293	Supported
H_5_	PC* Gender- > BT- > PI	0.002	0.265	Not supported
H_6_	PW* Gender- > BT- > PI	0.021*	2.009	Supported

PW = perceived competence; PC = perceived warmth; BT = brand trust; PI = purchase intention; PW* Gender = moderating effect of gender on perceived warmth; PC* Gender = moderating effect of gender on perceived competence; * *p* < 0.05, ** *p* < 0.01, *** *p* < 0.001.

## Results and Discussion

By using gender as a moderator and brand trust as mediator, this study explored brand perception’s effects on consumer purchase intention by executing two experiments and a path analysis. Findings of the study support hypotheses H_1_, H_2_, H_3_, H_4_, and H_6_, however, hypothesis H_5_ is not supported. The findings of the three studies show that brand perception exerts a significant and positive effect on purchase intention; additionally, the results reveal perceived competence and perceived warmth to have a significant positive effect on purchase intention. These findings were noted to be consistent with those of Xu et al. (2013); Zawisza and Pittard (2015), and Wang et al. (2017). Both perceived competence and perceived warmth constitute positive brand cognition among consumers and reflect consumers’ positive attitudes toward brands. Positive brand attitude can increase purchase intention. Accordingly, hypotheses H_1_ and H_2_ were supported.

As verified in three studies, consumers’ brand perception was noted to positively affect purchase intention, with brand trust serving as a mediator. Perceived warmth and perceived competence were determined to exert positive effects on brand trust (Kim et al., 2018), and brand trust was noted to further positively affect purchase intention (Sultan and Wong, 2019). Even after the effects of consumer involvement were controlled, the brand perception–purchase intention relationship was completely mediated by brand trust in Experiment 2 and path analysis, further verifying the robustness and credibility of the conclusion. The perceived competence and perceived warmth of a brand among consumers derive from their comprehensive cognition resulting from accumulated brand knowledge and personal experience; this can promote consumer confidence in brands’ competence and warmth and eventually evolve into consumer brand trust. Therefore, hypotheses H_3_ and H_4_ were observed to be supported by the experimental data.

Zawisza and Cinnirella (2010) found that the gender of advertising characters does not moderate brand perception’s influence that is exerted on purchase intention, but this influence is moderated by product involvement, technology anxiety, and self- versus other-focused (Zawisza and Pittard, 2015). Researchers believe that the gender of consumers is highly crucial in exploring the effect exerted by brand perception on purchase intention from the perspective of information processing and consumer psychology. This study shows that consumers’ gender do not moderate the effect exerted by perceived competence on the consumers’ purchase intention but could moderate the effect exerted by perceived warmth on the consumers’ purchase intention. For male consumers, perceived competence was presented as to exert a direct effect on the consumers’ purchase intention as well as an indirect effect, through brand trust, on such intention. For female consumers, findings show that perceived competence exerts only an indirect effect through brand trust. Therefore, for male and female consumers, perceived competence’s effect on purchase intention was significant and positive. In other words, no moderating effect of gender on perceived competence’s influence on purchase intention was noted. A consistent conclusion was found in the path analysis that gender has no significant moderating impact of perceived competence on purchase intentions. The competence cues can enhance consumer confidence in product performance alongside increasing brand trust and purchase intention. This may reject the role of gender differences in psychological mechanism. Findings show no gender difference in the influence of perceived competence on purchase intention. Therefore, hypothesis H_5_ was not supported. For male consumers, perceived warmth had no effect (direct or indirect) on purchase intention. For female consumers, perceived warmth was demonstrated to have only an indirect effect, through brand trust, on the consumers’ purchase intention; in other words, perceived warmth persuades only women but not men. Therefore, perceived warmth’s effects on purchase intention can be moderated by gender. A consistent conclusion was found in the path analysis that gender can significantly moderate the effect of perceived warmth on purchase intentions. From the perspective of information processing, women process information more meticulously than men, thus, the warm cues in the information are more easily captured by women (Darley and Smith, 1995; Papyrina, 2019). The warm cues can stimulate consumer’s emotional reaction; according to the empathizing–systemizing theory, such reaction is more likely to promote female consumers to generate brand trust and increase purchase intention. Therefore, hypothesis H_6_ was supported.

Consumer brand cognition includes brand perception, which represents consumers’ social judgments regarding a brand. This contributes to perceived warmth and perceived competence for a brand and generates brand trust, affecting consumers’ purchase intention. This study explores consumers’ psychological mechanisms that could underlie the effects of brand perception on purchase intention; findings show brand perception as a key antecedent to affecting consumers’ purchasing decisions. More so, the moderating effect of gender is examined in this process. Thus, this study applied and expanded on the stereotype content model for consumer behavior. The moderating effect of gender is of considerable significance to corporate marketing practice.

## Managerial Implications and Limitations

By examining how brand perception affects purchase intention, an enterprise can establish a suitable brand perception among consumers. The suggestions of the current study focus on two points:

First, enterprises with different strengths must shape appropriate brand perceptions among consumers through various marketing tools, such as advertising, after-sales services, and word-of-mouth communication to positively promote brand trust and purchase intention. From a quantitative perspective, perceived competence was demonstrated to exert a greater effect on purchase intention when compared with perceived warmth; the reason is that perceived competence had a greater regression coefficient. However, not all companies can outperform competitors. In such cases, brand warmth perception among consumers can also promote their purchase intention. Therefore, shaping the warmth perception of consumers can provide a new direction for most companies. These companies have no advantage in competence compared with their competitors, but they may actively assume social responsibility or establish positive brand relationships with consumers to gain a reputation as being warm (Johnson et al., 2018).

Second, warmth perceptions should be created among consumers to promote purchase intention if the target customers are women instead of men. More precisely, women’s products brands need to stimulate the emotional response of female consumers by creating a warm shopping atmosphere, marketing efforts that make female customers feel warm, and promoting warm brand content, which can gain the trust of female consumers and improve their purchase intentions. This is because the effect of warmth perception on purchase intention was noted to be significant only for female consumers. In other words, when companies shape consumers’ perceptions regarding brands or companies, the gender of the target customers should be considered because competence perception is effective for both male and female consumers but warmth perception is effective only for female consumers.

In this study, virtual brands were adopted in the experiments to overcome inherent stereotypes and consumer preferences for real brands. Nevertheless, in future studies, cooperation with real brands in field experiments could be included. In the current study, online experiments were used to improve the external validity of the sample. However, the study participants were not tested in a controlled environment and might have, therefore, been affected by the environmental variables. Future studies may conduct experiments with controlled environmental stimuli to validate the findings of the present study.

## Data Availability Statement

All datasets generated for this study are included in the article/Supplementary Material.

## Ethics Statement

The study was carried out in accordance with the recommendations of the Local Ethics Committee of Shenzhen University with written informed consent from all participants. All participants gave written informed consent in accordance with the Declaration of Helsinki. The protocol was approved by the Local Ethics Committee of Shenzhen University.

## Author Contributions

JX conceived, designed and performed the experiments, and made the manuscript modification. ZZ provided guidance for the experiments and assistance in the interpretation of the supplementary research related to SEM. LZ participated in the experimental implementation and wrote the draft of the manuscript. SM participated in the revisions, results and analysis of SEM models, and improving the manuscript language.

## Conflict of Interest

The authors declare that the research was conducted in the absence of any commercial or financial relationships that could be construed as a potential conflict of interest.

## Footnotes

1 https://www.wjx.cn/
