# Supplementary material for: Do Brand Competence and Warmth Always Influence Purchase Intention? The Moderating Role of Gender
Source: Front Psychol. 2020 Feb 21;11:248. doi: 10.3389/fpsyg.2020.00248 (PMC7046750; doi:10.3389/fpsyg.2020.00248)
Supplement: Supplementary file 1 [file Table_1.docx]

| **Discriminate validity analysis between constructs.** | | | | |
| --- | --- | --- | --- | --- |
|  | 1 | 2 | 3 | 4 |
| Brand trust | 0.872 |  |  |  |
| Perceived competence | 0.800 | 0.829 |  |  |
| Perceived warmth | 0.837 | 0.781 | 0.832 |  |
| Purchase intention | 0.837 | 0.745 | 0.768 | 0.903 |
| Note: numbers on the diagonal line represent the square root of the AVE value; other numbers represent the correlation coefficient. | | | | |
